# Costs of distributing HIV self-testing kits in Eswatini through community and workplace models

**DOI:** 10.1186/s12879-023-08694-y

**Published:** 2024-02-29

**Authors:** Kathleen McGee, Marc d’Elbée, Ralitza Dekova, Linda A. Sande, Lenhle Dube, Sanele Masuku, Makhosazana Dlamini, Collin Mangenah, Lawrence Mwenge, Cheryl Johnson, Karin Hatzold, Melissa Neuman, Gesine Meyer-Rath, Fern Terris-Prestholt

**Affiliations:** 1 Faculty of Public Health and Policy, London School of Hygiene and Tropical Medicine, London, UK; 2 Population Services International, Mbabane, Eswatini; 3 Malawi Liverpool Wellcome Trust Research Programme, Blantyre, Malawi; 4 Ministry of Health, Mbabane, Eswatini; 5 Centre for Sexual Health and HIV/AIDS Research, Harare, Zimbabwe; 6 Zambart, Lusaka, Zambia; 7 World Health Organisation, Global HIV, Hepatitis and STI Programmes, Geneva, Switzerland; 8 Population Services International, Cape Town, South Africa; 9 Faculty of Epidemiology and Population Health, London School of Hygiene and Tropical Medicine, London, UK; 10 Center for Global Heath and Development, Boston University School of Public Health, Boston, USA; 11 Health Economics and Epidemiology Research Office, Department of Internal Medicine, Faculty of Health Sciences, University of the Witwatersrand, Johannesburg, South Africa

**Keywords:** HIV, HIV self-testing, Costs and cost analysis, Community, Workplace, Eswatini

## Abstract

**Background:**

This study evaluates the implementation and running costs of an HIV self-testing (HIVST) distribution program in Eswatini. HIVST kits were delivered through community-based and workplace models using primary and secondary distribution. Primary clients could self-test onsite or offsite. This study presents total running economic costs of kit distribution per model between April 2019 and March 2020, and estimates average cost per HIVST kit distributed, per client self-tested, per client self-tested reactive, per client confirmed positive, and per client initiating antiretroviral therapy (ART).

**Methods:**

Distribution data and follow-up phone interviews were analysed to estimate implementation outcomes. Results were presented for each step of the care cascade using best-case and worst-case scenarios. A top-down incremental cost-analysis was conducted from the provider perspective using project expenditures. Sensitivity and scenario analyses explored effects of economic and epidemiological parameters on average costs.

**Results:**

Nineteen thousand one hundred fifty-five HIVST kits were distributed to 13,031 individuals over a 12-month period, averaging 1.5 kits per recipient. 83% and 17% of kits were distributed via the community and workplace models, respectively. Clients reached via the workplace model were less likely to opt for onsite testing than clients in the community model (8% vs 29%). 6% of onsite workplace testers tested reactive compared to 2% of onsite community testers. Best-case scenario estimated 17,458 (91%) clients self-tested, 633 (4%) received reactive-test results, 606 (96%) linked to confirmatory testing, and 505 (83%) initiated ART.

Personnel and HIVST kits represented 60% and 32% of total costs, respectively. Average costs were: per kit distributed US$17.23, per client tested US$18.91, per client with a reactive test US$521.54, per client confirmed positive US$550.83, and per client initiating ART US$708.60. Lower rates for testing, reactivity, and linkage to care in the worst-case scenario resulted in higher average costs along the treatment cascade.

**Conclusion:**

This study fills a significant evidence gap regarding costs of HIVST provision along the client care cascade in Eswatini. Workplace and community-based distribution of HIVST accompanied with effective linkage to care strategies can support countries to reach cascade objectives.

**Supplementary Information:**

The online version contains supplementary material available at 10.1186/s12879-023-08694-y.

## Background

With an estimated HIV prevalence of 27% in 2019, Eswatini has the highest HIV prevalence in the world [1]. In 2020, an estimated 210,725 persons in Eswatini lived with HIV, of whom 63.4% were female [2]. The number of new HIV infections have significantly declined over the last decade, with annual incidence rates decreasing from 17.24 per 1000 in 2011 to 4.9 per 1000 in 2019 [1]. With a population close to 1.1 million people [2], a robust national implementation of “Test and Treat”, and targeted prevention for young populations, Eswatini edges closer to achieving UNAIDS’ 95-95-95 targets, and by extension, epidemic control. As of 2019, an estimated 93% of people living with HIV (PLHIV) knew their status, 86% of PLHIV in Eswatini were on treatment, and 80% of PLHIV on treatment were virally suppressed [2]. The country’s immediate goal is to identify the remaining portion of PLHIV who are unaware of their status and link them to care.

For case identification of both undetected and new infections, innovative approaches must be exploited. HIV self-testing (HIVST) is one such approach, recommended by the World Health Organization (WHO) [3]. Demonstrated as both feasible and acceptable across various research settings [4–9], HIVST may help remove common barriers known to provider-delivered HIV testing services (HTS) such as stigma, discrimination, and distance to testing centres [10]. HIVST have been adopted in Eswatini as a supplementary approach to standard HTS in order to improve case finding among hard-to-reach populations, specifically men, young people, and key populations [3]. Both assisted and unassisted approaches have been adopted, and different models are currently being investigated in-country by various implementing partners. Since 2017, Population Services International (PSI), along with the technical support and funding of the HIV Self-Testing AfRica Initiative (STAR), has been distributing HIVST across Eswatini, using both community and workplace distribution models.

The combination of HIVST technology and different distribution strategies may generate a significant public health impact and support Eswatini in closing the 95-95-95 gap. However, at its lowest average kit cost of US$2 *ex works* (price not including delivery, distribution, taxes or commission charges) in the public sector for low- and middle-income countries (LMIC) [11], the HIVST kit remains twice as expensive as a standard HIV rapid diagnostic test (RDT) [12]. In a context of scarce resources, policymakers and funding partners need further evidence on the costs of distributing HIVST. This costing study aims to fill this gap, by estimating total and average costs of the community-based and workplace HIVST distribution models, and present costs along the HIV cascade of care, i.e. per person tested for HIV, per person identified positive, and per person effectively linked to care. Each of these steps moves closer to achieving the ultimate health outcome of healthier and longer lives for PLHIV.

## Methods

### Setting and intervention

Between April 2019 and March 2020, Population Services International (PSI) distributed oral fluid-based HIVST kits (OraQuick HIV-1/2 Rapid Antibody Test) across Eswatini to youth, defined as persons between 16–24 years of age, adult men and women, defined as 25–49 years, and key populations, including sex workers (SW), men who have sex with men (MSM), and injection drug users (IDU). Note that in 2019, the age of consent for HIVST was 16 and above. In this period, PSI used the following distribution models:

- **Community-based model**: This model consisted of multiple approaches, including kit distribution at community-wide events, popular community spots, and through door-to-door campaigns. Targeting large communal venues, this model aimed to rapidly increase testing coverage among adult and adolescent women and men (16–49 years) who may not present to health facilities for conventional HIV testing with a health provider. This model targeted areas with reported high prevalence, covering key population hotspots for female and male SW, MSM, and IDU.
- **Workplace model**: Kits distribution took place in courtyards of industrial worksites with employees numbering up to 300 individuals. This approach targeted industrial areas populated by manufacturing plants in the city and aimed to increase testing coverage among population subgroups typically neglected by conventional HTS due to work commitments. Target populations included adult men and women aged 16 to 49 years and their sexual partners and families. This model aligned to the Ministry of Health’s strategy for HIVST which included distribution in male-dominated workplaces.

HIVST distributors comprised of PSI-salaried officers and volunteer health workers typically engaged in the community model, as well as wellness officer nurses dedicated to the workplace model. Distributors received training on the mechanics of oral HIVST kits, assessing clients’ HIV risk profile, screening for intimate partner violence, responding to frequently asked questions, and conducting telephone follow-up calls with HIVST kit recipients.

**Fig. 1:**
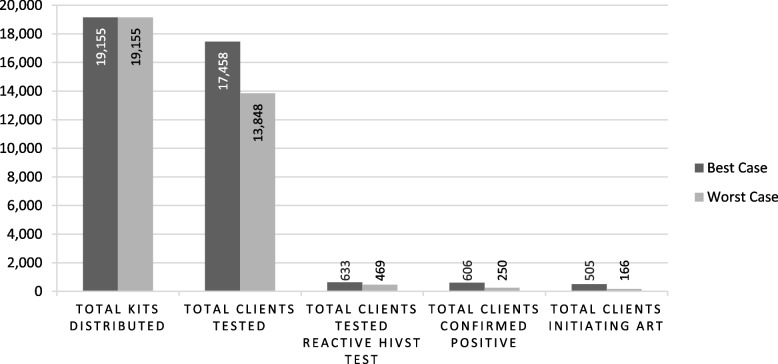
HIV self-testing care cascade results per best-case and worst-case estimates

In both models, clients were screened by HIVST distributors using a Ministry of Health-prescribed risk assessment tool comprising of seven clinical and behavioural questions to evaluate clients’ HIV status, date of last HIV test, and recent risk exposure. Clients could choose to self-test onsite, take a kit for offsite self-testing, and/or take a kit to a partner or friend (secondary distribution). For onsite-testing, clients were provided with pre-test counselling before choosing to self-test with or without assistance. Testing took place in a private room or gazebo, pre-equipped with an instructional video and materials. Clients were encouraged to disclose testing results and, if tested reactive, referred to HIV confirmatory testing and services. To confirm test results, the HIV diagnostic algorithm in Eswatini entails two provider-administered blood-based HIV antibody tests; should both tests be reactive, the client is diagnosed HIV positive. Clients choosing to self-test offsite received pre-test counselling and a screening for risk of intimate partner violence before being offered a self-test kit and an instructional video shared through WhatsApp, along with a flyer for further information. Offsite clients were encouraged to call a toll-free hotline for support. In case of a reactive or inconclusive test result, clients were advised to link to a healthcare facility for confirmatory testing and treatment. Clients who tested negative were directed towards HIV prevention services. Clients were additionally offered self-test kits to distribute to a person of their choosing, such as a sexual partner, IDU partner, family member, or friend. In such cases, clients were provided with an instructional video and hotline number via WhatsApp to share with a secondary client. The video contained key messages regarding linkage to care for both reactive and non-reactive clients. A client flow-chart illustrating various service options is presented in Fig. 1, Additional file 1.

### Study design and data collection

We conducted a top-down incremental cost analysis of HIVST kit distribution in Eswatini across community and workplace models. This study estimates the programme’s economic costs over 1 year, from April 2019 to March 2020, from the provider perspective. We estimated the total costs of HIVST kit distribution per model, and average cost per HIVST kit distributed along the client care cascade.

In this study, the provider was principally PSI/Eswatini. PSI-salaried officers distributed the majority of kits (89%), while volunteers (9%) and government nurses (2%) distributed remaining kits. Volunteers were compensated SZL 100 (US$6.66) a day, and nurses were paid a monthly salary of SZL 22,639 (US$1,508.26) by private-sector companies. Public health facilities covered the ensuing costs of confirmatory testing and anti-retroviral therapy (ART) initiation. These additional contributions were included in the analysis to reflect the program’s total economic costs. Due to limited data on the average costs of confirmatory testing and ART initiation in Eswatini and other low-income countries, these were estimated at US$6 and US$55 respectively, as recently evaluated in South Africa [13, 14].

This study evaluated costs from April 2019 to March 2020, capturing the implementation costs across a period of 12 months, but excluding the program’s start-up costs incurred in 2018. From October 2018 to March 2019, PSI-Eswatini suffered a 6-month program interruption, and from March 2020 onwards, field activities were adapted to respond to COVID-19. Furthermore, the program’s start-up period was marked by high staff turnover and alternating distribution models, thus confounding analysis. To facilitate generalizability of study findings, the period of analysis was selected to reflect best the program’s routine costs.

Using a top-down costing approach, project expenditures were collated from PSI’s financial reports, complemented by interviews with key project personnel. Each line item was categorized according to input category and then apportioned to each model using predefined allocation factors (Table 1, Additional file 2), such as number of tests distributed, number of persons trained, or staff time dedicated per each model. Research costs such as researcher salary costs were excluded. Costs incurred in local currency (Swazi Lilangeni or SZL) were inflated to 2020 using the gross domestic product (GDP) deflator in local currency [15], and then converted to US dollars using the 2020 exchange rate [16]. Training and sensitization were annualized using 2 years of assumed useful life, other capital costs 5 years, and a 3% discount rate was applied, following international costing guidelines [17].

**Table 1:** Summary of distribution, onsite testing, and offsite interview outcomes

**HIVST Distribution and Primary Client Data per Distribution Model, April 2019 to March 2020**				
	**Community** **N (%)**	**Workplace** **N (%)**		**Total**
**Total # of tests distributed**	15,864 (83%)	3291 (17%)		19,155
**Total # of clients reached**	11,091 (85%)	1932 (15%)		13,031
**Average # of tests per client**	1.43	1.70		1.47
**Onsite HIV Self-Testing Results per Distribution Model, April 2019 to March 2020**				
	**Community** **N (%)**	**Workplace** **N (%)**		**Total** **N (%)**
**Total # of clients reached**	11,091	1932		13,023
**On/offsite Testing**				
**Did not test on site**	7929 (71%)	1784 (92%)		9713 (75%)
**Tested on site**	3162 (29%)	148 (8%)		3310 (25%)
**Onsite Testing Results**				
**Reactive**	67 (2%)	9 (6%)		76 (2%)
**Non-reactive**	3055 (97%)	136 (92%)		3191 (96%)
**Results non-disclosed**	40 (1%)	3 (2%)		43 (1%)
**Results of Follow-up Calls with HIVST Primary Clients**				
	**Community** **N (%)**	**Workplace** **N (%)**	**Unknown** **N (%)**	**Total** **N (%)**
**Total # of primary clients followed-up**	687	234	1073	1994
**Reported use of HIVST kit (*n* = 1994)**				
**Yes**	572 (83%)	196 (84%)	591 (55%)	1359 (68%)
**No**	29 (4%)	7 (3%)	37 (3%)	73 (4%)
**NA/No Response**	86 (13%)	31 (13%)	445 (41%)	562 (28%)
**Reported HIVST result (*n* = 1395)**				
**Reactive**	24 (4%)	4 (2%)	37 (6%)	65 (5%)
**Non-reactive**	541 (95%)	186 (95%)	537 (91%)	1264 (93%)
**Unknown/No Response**	7 (1%)	6 (3%)	17 (3%)	30 (2%)
**Confirmed testing (*n* = 65)**				
**Yes**	15 (63%)	1 (25%)	15 (41%)	31 (48%)
**No**	1 (4%)	-	1 (3%)	2 (3%)
**Unknown/No Response**	8 (33%)	3 (75%)	21 (57%)	32 (49%)
**Confirmed HIV result (*n* = 31)**				
**Positive**	14 (93%)	1 (100%)	13 (87%)	28 (90%)
**Negative**	-	-	-	-
**Unknown/No Response**	1 (7%)	-	2 (13%)	3 (10%)
**Initiated ART (*n* = 28)**				
**Yes**	9 (64%)	1 (100%)	7 (54%)	17 (61%)
**No**	2 (14%)	-	2 (15%)	4 (14%)
**NA/No Response**	3 (21%)	-	4 (31%)	7 (25%)
**Total # of Secondary clients**	344	135	324	803
**Reported use of HIVST kit (*n* = 803)**				
**Yes**	205 (60%)	80 (59%)	228 (70%)	513 (64%)
**No**	40 (12%)	25 (19%)	60 (19%)	125 (16%)
**NA/No Response**	99 (29%)	30 (22%)	36 (11%)	165 (21%)
**Reported result of HIVST (*n* = 513)**				
**Reactive**	2 (1%)	1 (1%)	7 (3%)	10 (2%)
**Non-reactive**	185 (90%)	63 (79%)	207 (91%)	455 (89%)
**NA/No Response**	18 (9%)	16 (20%)	14 (6%)	48 (9%)
**Confirmed HIV result (*n* = 10)**				
**Positive**	1 (50%)	-	2 (29%)	3 (30%)
**Negative**	-	-	-	-
**NA/No Response**	1 (50%)	1 (100%)	5 (71%)	7 (70%)
**Initiated ART (*n* = 3)**				
**Yes**	-	-	-	-
**No**	-	-	-	-
**NA/No Response**	1 (100%)	-	2 (100%)	3 (100%)

Based on PSI headquarters’ procurement reports. the average cost per kit, including purchasing and freight costs, was US$3.11. Economic costs (other costs not reflected in PSI’s expense reports), such as personnel costs paid for by local partners, were collected from interviews with the HIVST project manager.

Program outcomes were directly collected from PSI’s monitoring and evaluation (M&E) data. Field agents prospectively collected data via paper-based tools, then entered into Microsoft Access by data clerks. Tools developed for data collection, quality assurance, and training of field agents were revised and endorsed by Eswatini’s National AIDS Programme. Field agents received start-up and routine training and supervision from PSI’s M&E manager and HIVST manager. PSI maintained two databases to track project outcomes. The Distribution Tracker monitored the number of kits distributed across models and geographic regions and recorded demographic information and testing results of onsite testing clients. Onsite test results were only recorded when voluntarily disclosed by clients. Clients who opted to test offsite were offered telephonic follow-up services by PSI. Clients were asked for their phone number and their consent for follow-up calls. Non-consenting individuals were not contacted. Within 1 month of distribution, PSI selected one-third of consenting clients and attempted to contact each client up to three times to collect information on testing results and follow-up care. Clients were also asked to report if they provided kits to secondary recipients and disclose secondary testing results if known. Project data was collected and maintained by PSI’s M&E team and then shared with this study’s author after removing data identifying specific individuals.

To estimate average costs, total program costs were disaggregated by model and then divided by program outcomes. This study estimates the average cost per kit distributed, per client tested, per client tested reactive, per client confirmed positive, and per client initiating ART. Total kits distributed were directly calculated from reported M&E data. All other outcomes, such as total tested, total reactive, total confirmed positive, and total initiating ART, were estimated using respondents’ self-reported rates in follow-up interviews.

### Sensitivity and scenario analyses

One-way sensitivity analysis was used to assess the impact of key cost assumptions on average costs. The 3% discount rate used to annualize costs was varied from 0% to 6.5%, to capture the effects of either using no discount rate or using Eswatini’s national interest rate during project implementation (6.5% in 2019) [18]. To evaluate the effects of assumed life years on capital costs, annualization was varied between 1 and 3 years for training and sensitization (base case of 2 years), and 2.5 and 7.5 years for equipment costs (base case of 5 years). Costs per HIVST kits were varied to a minimum of US$1 to reflect the current price of a finger-prick rapid diagnostic test [12], and a maximum of US$3.46 ex works to reflect the kit’s most expensive unit cost during the project life. Finally, headquarter and field personnel costs were varied by ±10% to assess the impact of salaries on overall distribution costs.

Scenario analysis was conducted to explore the impact of key epidemiological assumptions made when estimating health outcomes on our results. Clients who were not contacted for follow-up interviews (*n* = 7,719), otherwise considered as lost-to-follow-up (LTFU), and clients contacted for follow-up but declined to respond (*n* = 562), henceforth referred to as non-responders, were assumed to adopt the same testing behaviours as respondents. Best-case scenario estimated health outcomes assuming all clients, including non-responders and persons LTFU, shared similar testing behaviours as responders. The worst-case scenario assumed non-responders did not self-test or further engage in care, resulting in lower estimates of testing uptake, reactivity rate, and linkage to care. Best-case estimates may be affected by responder and social-desirability bias, while worse-case estimates are affected by a substantial non-responder bias of over 28%. The true estimates, while remaining unknown, are assumed to lie in between both scenarios.

In consideration of the often fluctuating and generally declining yields seen across HIV testing programs as higher coverage is reached, an additional scenario analysis was conducted exploring the effects of alternative reactivity rates on average costs. Reactivity rates were varied from 6%, reflecting PEPFAR’s reported average testing yield in Eswatini in 2018 [2] to an-assumed minimum of 1%.

## Results

### HIVST kits distribution and follow-up survey

From April 2019 to March 2020, PSI distributed a total of 19,155 HIVST kits to a total of 13,023 primary clients, averaging 1.5 kits distributed per recipient. Kits were nationally distributed, 83% by the community model, and 17% by the workplace model. Most primary clients were female (53%), mean age was 29 (Standard Deviation 8.9), and 28% reported having not tested for HIV in a year (See Table 1 for summary results, see Additional file 3 for additional demographic details).

A quarter of clients chose to self-test onsite (*n* = 3310/13023, 25%), though 21 per 100 additional persons opted for onsite self-testing in the community model compared to the workplace model (*n* = 3162/11091 or 29% vs. *n* = 148/1932 or 8%). Among onsite testers, 76 recipients (2%) had a reactive self-test. Onsite workplace testers had 4 additional reactive cases per 100 people tested compared to onsite community testers (*n* = 9/148 or 6% vs. *n* = 67/3162 or 2%).

Among remaining clients who took a kit home for personal use and/or secondary distribution (*n* = 9713), 1,994 (21%) were contacted by M&E officers. Non-response rate was high at 28% (*n* = 562/1994). Among respondents (*n* = 1432), 1,359 (95%) clients reported self-testing, among whom, 65 (5%) reported a reactive self-test result. 31 of 33 (94%) remaining responders confirmed seeking confirmatory testing, and 17 of 21 (81%) responders reported initiating ART.

Among clients who distributed their tests onwards and reported on secondary distribution (*n* = 638/1432, 45%), 513 (80%) reported secondary clients had used their HIVST kit. 49% of secondary clients were sexual partners, 23% friends, 16% family members, and 8% biological children. 10 secondary reactive cases were known and reported by primary clients and three were known to have sought confirmatory testing. No respondent knew whether a secondary recipient of an HIVST kit had initiated ART.

Total implementation results for onsite testers, offsite primary clients, and offsite secondary clients were estimated using best-case and worst-case assumptions. See Additional file 4 for details on scenario estimates, measurements, and assumptions. In the best-case, total testing uptake was estimated at 91% (*n* = 17,458/19155), reactivity rate at 3.62% (*n* = 633/17458), linkage to confirmatory testing at 96% (*n* = 606/633), and ART initiation at 83% (*n* = 505/606). Due to high non-response rates, worst-case estimated testing uptake at 72% (*n* = 13,458/19155), reactivity rate at 3.4% (*n* = 469/13458), confirmatory testing at 53% (*n* = 250/469), and ART initiation at 66% (*n* = 166/250).

### Cost analysis

Total program costs amounted to US$330,069.60, of which 83% were allocated to the community distribution model and 17% to the workplace model (Table 2). Average cost was US$17.23 per kit distributed, US$18.91 per client tested, and US$521.54 per client tested reactive. Including the costs of linkage to care in a facility, the average cost per client confirmed positive was estimated US$544.83 and per client initiating ART at US$653.60. Local personnel and HIVST kits represented 41% and 32% of total costs, respectively.

**Table 2:** Total HIV self-testing costs per model and average costs along the client care cascade (in 2020 US$)

	**Community**		**Workplace**		**Total**	
	**Costs**	**%**	**Costs**	**%**	**Costs**	**%**
Input types						
***Start-up***						
Training	$366	0%	$47	0%	$413	0%
Sensitization	$482	0%	$482	1%	$963	0%
***Capital***						
Building & storage	$2,852	1%	$592	1%	$3,444	1%
Equipment	$502	0%	$104	0%	$606	0%
*Start-up and Capital - sub-total*	*$4,202*	2%	*$1,225*	2%	$5,427	2%
***Recurrent***						
Personnel & Per diems	$164,821	60%	$34,181	13%	$199,003	60%
Volunteers & Per diems	$313	0%	$200	0%	$513	0%
HIVST Kits	$86,373	32%	$17,920	31%	$104,293	32%
Other Supplies (excluding HIVST kits)	$2,143	1%	$445	1%	$2,588	1%
Vehicle operation, maintenance & transport	$6,158	2%	$ 1,616	3%	$7,774	2%
Building operation/maintenance	$1,877	1%	$389	1%	$2,266	1%
Other recurrent	$6,796	2%	$1,410	2%	$8,206	2%
*Recurrent - sub-total*	*$268,482*	98%	*$56,161*	98%	$324,643	98%
**Total HIVST costs**	***$272,684***		***$57,386***		**$330,070**	
**Cost per kit distributed**	**$17.19**		**$17.44**		**$17.23**	
**Cost per client self-tested**	**$18.77**		**$19.56**		**$18.91**	
**Cost per client self-tested reactive**	**$526.86**		**$497.69**		**$521.54**	
**Cost per client confirmed HIV positive**^**a**^	**$556.34**		**$526.10**		**$550.83**	
**Cost per client initiating ART**^**b**^	**$713.54**		**$685.04**		**$708.60**	

*HIVST* HIV self-testing, *ART* Antiretroviral treatment

^a^Includes estimated $6 cost of facility-based confirmatory testing with rapid tests [13]

^b^Includes estimated $55 cost of an ART initiation visit [14]

Additional files 5 and 6 plot this study’s average cost per HIVST kit distributed and average cost per person tested along with number of tests provided. Furthermore, these graphics compare this study’s results to other HIVST and HTS costing studies conducted in sub-Saharan Africa.

### Sensitivity and scenario analyses

**Fig. 2:**
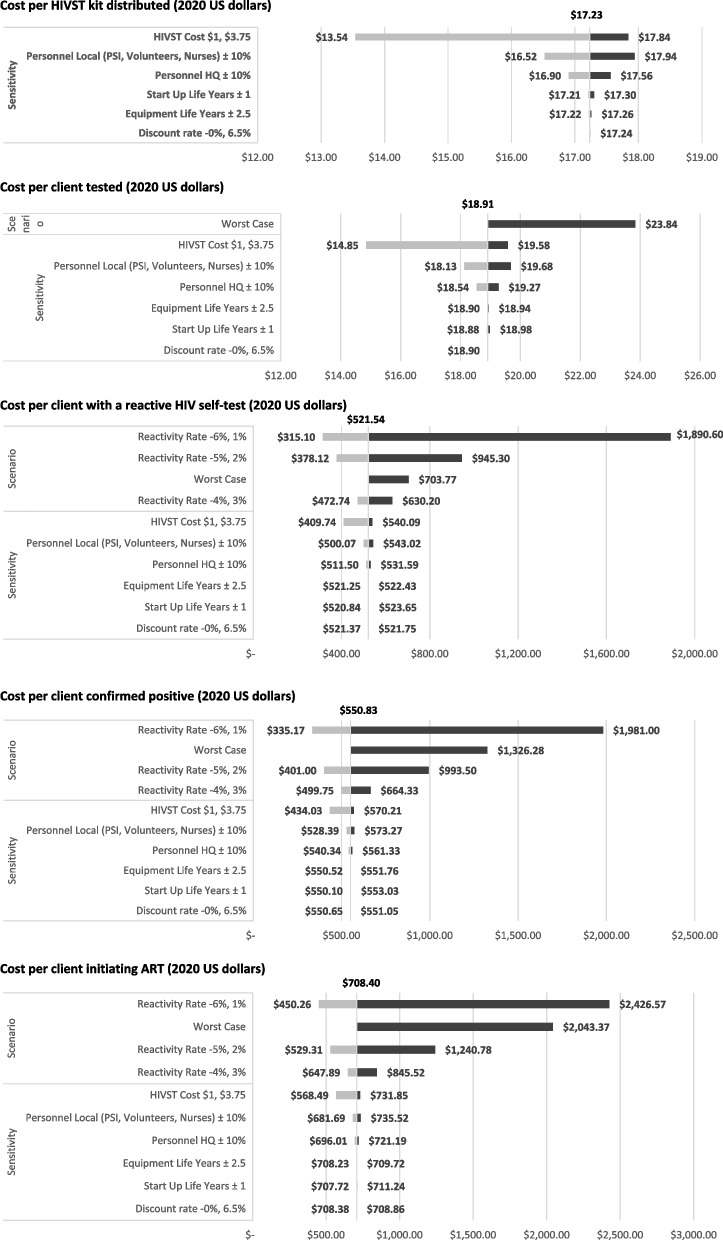
Sensitivity and Scenario analyses (2020 US dollars)

Results of the univariate sensitivity and scenario analyses are presented in Fig. 2. Average costs along the client care cascade remained robust in the sensitivity analysis. Variations to assumed life years, discount rate, and HQ personnel costs resulted in minimal changes to results, while variations of ±10% to field personnel costs and changes to average price per HIVST kit yielded stronger effects on average costs.

Scenario analyses yielded the greatest changes on average costs. The worst-case scenario, resulted in higher average costs per client tested (US$23.96), per client tested reactive (US$542.91), per client confirmed positive (US$573.15), and per client initiating ART (US$735.20). Decreasing reactivity rates resulted in significant increases to the average costs per client tested reactive (US$315.10-US$1,890.60), per client confirmed positive (US$335.17-US$1,981), and per client initiating ART (US$450.26-US$2,426.57).

## Discussion

Distribution of HIVST kits across community and workplace models resulted in high testing coverage and case identification. The community model served as an expansive approach dispensing kits to targeted areas of high HIV prevalence and populations at higher risk of HIV infections. The workplace model targeted populations assumed to be likely neglected by conventional HTS approaches. Neither model distinctly helped to reach men, young people and key populations as stipulated in program goals: over half of clients were female (53%), over 60% were over the age of 25, and 99% were recorded as general population, indicating further effort is needed to reach these target demographics. Testing behaviours of each population were different, with clients reached through the community model more likely to opt for onsite testing than clients reached through the workplace model. Workplace clients may be more reluctant to choose onsite HIV testing due to fear of stigma from co-workers and potential loss of employment, echoing previous findings documenting the role of stigma in deterring conventional HTS uptake [10]. Among workplace clients who tested onsite, 6% tested reactive compared to 2% of community clients, indicating the model may be a promising approach for case-identification, targeting populations otherwise neglected by standard services due to work commitments. Preliminary data from South Africa on workplace HIVST distribution found high HIVST uptake among a male population that had never previously tested for HIV [19]. Combined, these two studies reveal workplace distribution of HIVST may effectively target underserved populations and unidentified HIV cases.

This study’s estimated HIVST distribution costs along the treatment cascade must be discussed in the context of Eswatini’s achievements and the remaining work necessary towards ending the country’s AIDS epidemic. Despite having the greatest prevalence rate in the world, Eswatini has surpassed its first 90-target, leaving a small albeit hard-to-reach population of remaining cases. In such context, where testing volumes continue to decline, costs per test, per person tested positive, and per person initiated to ART will respectively increase. Targeted approaches, including workplace and recurring HIVST community-distributions, are essential to help complement Eswatini’s national strategies.

At an average cost of US$17.23 per kit distributed and US$18.91 per person self-tested, this study found HIVST distribution costs comparable to other HIVST costing studies. Community HIVST distribution in Malawi, Zambia, and Zimbabwe has been estimated (adjusted to 2020 US dollars) at US$6.56, US$13.46, and $14.23 per kit distributed, respectively [20], US$9.21 in Zambia [21], US$14.00 in Lesotho [22], and US$4.30-US$4.35 in South Africa [19]. As seen in other contexts [22], HIVST distribution efficiency may improve over time, potentially leading to lower average costs per kit distributed. Generalizability of study findings to different contexts and HIVST programs should be approached with consideration for local circumstances and adapted appropriately. Costs of field personnel, which account for 42% of running costs in our study, may dramatically vary across programs, with volunteer distributors costing significantly less than professional HTS counsellors. In some studies, HIVST distribution was integrated into the delivery of HTS services [22]. In South Africa, some distribution targeted high-traffic hotspots (e.g., taxi and train stations), reaching economies of scale [23]. Indeed, prior studies reporting lower unit costs had distributed upwards of 100,000 kits [20, 21, 23], representing over five times the distribution volume seen in Eswatini (Additional file 5). Indeed, countries with higher distribution volumes bear greater number of new annual HIV infections, further intimating that the smaller and more targeted the demand the greater the unit cost.

At US$18.91, the average cost per person tested is comparable to community-based HIV testing services in sub-Saharan African countries. A recent systematic review of the costs of HTS across sub-Saharan Africa has estimated the average cost per person tested in 2020 between US$16.47 in home-based HTS and $27.64 in campaign-style HTS (Ahmed N, Terris-Prestholt F, Ong JJ, et al. A systematic literature review of costs and cost-effectiveness analyses of HIV testing services in sub-Saharan Africa, forthcoming). In Eswatini, estimates have ranged from US$7.96-US$9.65 per person tested in health facilities to US$19.68 per person tested via community-based HTS [24–26]. In any given year, HTS services typically reach only a fraction of the numbers reached via HIVST, as evidenced in Additional file 6. Albeit slightly more expensive than the standard of care, HIVST remains an attractive supplementary approach to expanding testing coverage, reaching populations less likely to present at facilities and possibly earlier in the disease stage. As the WHO recommends countries transition to a 3-test strategy in facilities to ensure accurate diagnosis and to achieve high positive predictive value, HIVST may play a new role supporting countries to implement verification testing efficiently, while maintaining quality, and saving costs [27, 28].

Due to the private nature inherent to HIVST, only one other study has reported, to date, on the average costs of HIVST-distribution along the remainder of the care cascade. Measured across eleven different distribution models in South Africa, the average cost per client screened reactive was estimated between U$24-US$2,258, per client confirmed positive between US$52-US$7,345, and per client initiating ART between US$104-US$7,883. Eswatini’s cost estimates fall within those ranges, but the high heterogeneity in those results and this study’s scenario analyses demonstrate the importance of yield rates and linkage to care (LtC). As Eswatini nears its 95-95-95 targets, finding unidentified cases will become increasingly more challenging, raising total costs and average costs along the client cascade. Costs will further rise if LtC is not assured. In contexts like Eswatini, where program goals are to expand testing coverage and to diagnose and treat PLHIV, HIVST distribution must be accompanied by effective LtC strategies. While this study measures costs from the provider’s perspective, it is important to note that community and workplace distribution of HIVST kits relieves clients’ from direct and indirect costs of testing at a health facility [29]. However, the societal costs for clients seeking confirmatory testing and ART initiation remain, and programs seeking to encourage LtC could explore various ways to reduce the client’s financial burden, and by extension, barrier to care by offering, for example, immediate and community-based confirmatory testing and ART services [30–32].

Sensitivity analysis highlighted potential cost-saving opportunities. When HIVST kits were valued at the same price as a finger-prick test, costs per kit distributed fell as low as $13.62, and subsequent cascade unit costs fell by over 20%, a particularly encouraging finding as HIVST kit costs decline. Field-based personnel contributed over 40% of total costs, suggesting that local volunteers may serve as a cost-saving alternative to professional distributors. This approach, however, may require additional expenses for monitoring, quality assurance, and capacity building of the volunteer workforce. Other cost-minimization approaches may explore integrating HIVST into existing community health services [33], using existing infrastructures, resources, and personnel to deliver HIVST. While this may help minimize costs, overly relying on existing HTS infrastructures may limit the strategy’s potential in reaching underserved communities and need careful considerations for budgeting [22].

This study has limitations. As previously discussed, both best-and-worst-case scenarios are defined based on assumptions. The worst-case scenario presents conservative results due to a high proportion of non-responders, and the best-case scenario presents overly optimistic results, compromised by potential social-desirability bias. In presenting both scenarios, this paper attempts to overcome these biases, assuming true results lie within scenario estimates. Another study limitation is that reported reactive cases were not necessarily newly diagnosed. Moreover, a large portion of follow-up interviews did not collect client’s data, prohibiting a potentially interesting analysis on the demographic profile of reported reactive cases. Neither health outcomes nor costs were analysed at site level, and a geographic analysis of the data may provide further insight on cost variances between rural and urban areas. Due to the study’s selected time period, the costing precludes intervention start-up costs and only presents routine HIVST distribution costs. This study utilizes estimates from South Africa to measure cost of confirmatory testing and ART initiation due to lack of existing data for Eswatini in the literature.

Further research is needed to identify cost-minimization strategies without undermining health outcomes. Implementers should continue to explore the workplace model across various settings, populations, and prevalence-areas. Additional research is needed to compare, evaluate, and identify effective LtC interventions. Distribution programs should explore ways to reduce barriers to LtC including stigma, discrimination, and societal costs. Despite additional costs, if LtC can be ensured for HIVST clients, HIVST may likely become a more cost-effective approach. Finally, HIVST distribution costs and effects should be further studied under the lens of prevention, particularly in such settings as Eswatini where number of people with unknown status continue to decline.

## Conclusion

Study findings suggest that workplace and community-based distribution of HIVST, when accompanied by effective linkage to care strategies, can help countries achieve their cascade objectives. Estimated costs per kit distributed and per person tested are comparable to other studies and community-based HIV testing services in sub-Saharan African countries. However, as Eswatini achieves its 95-95-95 targets, the challenge of finding unidentified cases may increase total and average costs along the client cascade, emphasizing the importance of assuring effective linkage to care to mitigate rising expenses.

### Supplementary Information

**Additional file 1.** Client flow chart across community and workplace distribution models.

**Additional file 2.** Input categories and allocation factors.

**Additional file 3.** Distribution, onsite testing, and reported outcomes.

**Additional file 4.** Best-case and worst-case estimates, measurements, and assumptions.

**Additional file 5.** Average cost per HIVST kit distributed.

**Additional file 6.** Average cost per person tested in Eswatini by testing type.

## Data Availability

All data generated and analysed during this study are available from the corresponding author on reasonable request.

## Abbreviations

AIDS: Acquired immune deficiency syndrome
ARV: Anti-retroviral
ART: Anti-retroviral therapy
GDP: Gross Domestic Product
GHCC: Global Health Cost Consortium
HIV: Human immunodeficiency virus
HIVST: HIV self-testing
HTS: HIV testing services
IDU: Injection drug user(s)
LSHTM: London School of Hygiene and Tropical Medicine
LtC: Linkage to care
LTFU: Lost to follow up
M&E: Monitoring and evaluation
MOH: Ministry of Health
MSM: Men who have sex with men
NR: Non responders
PLHIV: People living with HIV
PrEP: Pre-exposure prophylaxis
PSI: Population Services International
RDT: Rapid diagnostic test
SOC: Standard of care
SOP: Standard operating procedure
STAR: HIV Self-Testing AfRica
SW: Sex worker
SZL: Swazi Lilangeni
UNAIDS: Joint United Nations Program on HIV/AIDS
VMMC: Voluntary Medical Male Circumcision
WHO: World Health Organization
