# Supplementary figures and images for: Costs of distributing HIV self-testing kits in Eswatini through community and workplace models
Source: BMC Infect Dis. 2024 Feb 29;22(Suppl 1):976. doi: 10.1186/s12879-023-08694-y (PMC10902928; doi:10.1186/s12879-023-08694-y)

**Additional file 1 – Client Flow Chart across community and workplace distribution models**

**
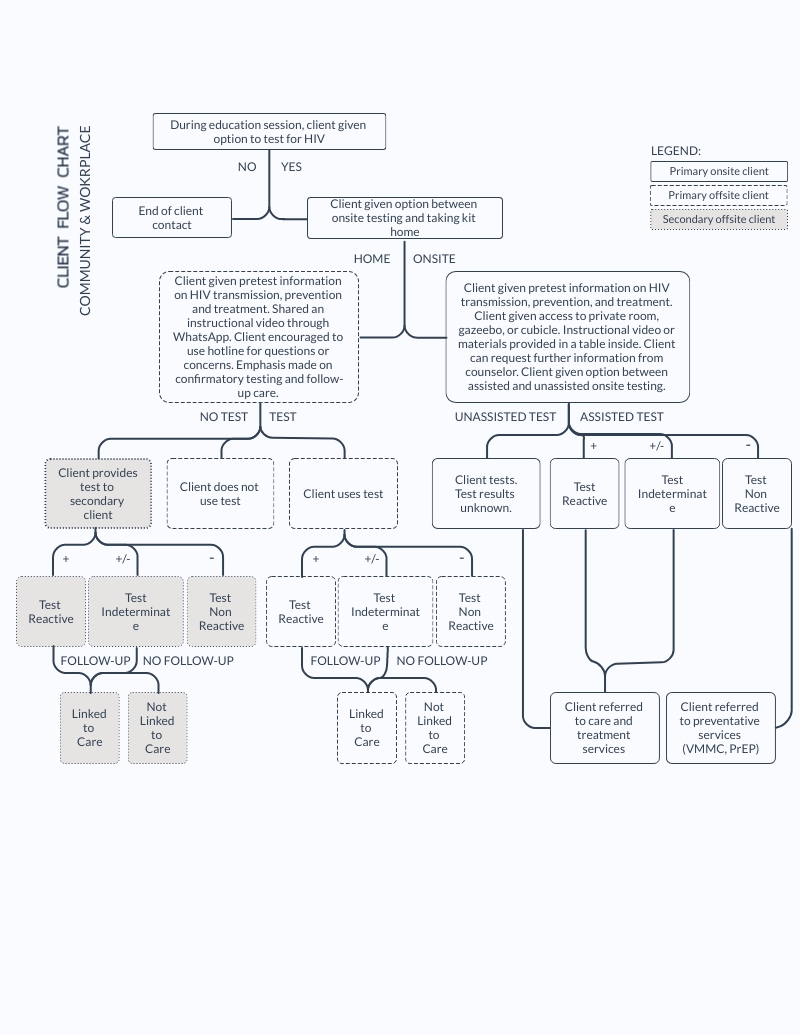
**

Supplement: Supplementary file 1 — Additional file 1. Client flow chart across community and workplace distribution models. [file 12879_2023_8694_MOESM1_ESM.docx]
