# Supplementary material for: Costs of distributing HIV self-testing kits in Eswatini through community and workplace models
Source: BMC Infect Dis. 2024 Feb 29;22(Suppl 1):976. doi: 10.1186/s12879-023-08694-y (PMC10902928; doi:10.1186/s12879-023-08694-y)
Supplement: Supplementary file 2 — Additional file 2. Input categories and allocation factors. [file 12879_2023_8694_MOESM2_ESM.docx]

**Additional file 2 – Input categories and allocation factors**

| Category | Input Category | Cost Descriptions | Expense Type | Useful Life | Allocation Factors to models | allocation factor % | Rationale |
| --- | --- | --- | --- | --- | --- | --- | --- |
| Start-up | **Training** | Program and M&E related trainings | Capital | 2 | % of participants per training | CM: 89%  FM: 0%  WM: 11% | # of participants proxy for time, space, and other training costs |
|  | **Sensitization** | Advertisements, media production and placement | Capital | 2 | Equally across models | CM: 33%  FM: 33%  WM: 33% | Sensitization messages relevant to all three models of distribution |
|  | **Other Start-up Costs** | None | - | - | - | - | - |
| Equipment | **Building and Storage** | Office and warehouse rent | Recurrent | 0 | % of kits distributed | CM: 82.71%  FM: 0.13%  WM: 17.16% | Kits distributed per model proxy for office and warehouse use |
|  | **Equipment** | Furniture, equipment, and software | Capital | 5 | % of kits distributed | CM: 82.71%  FM: 0.13%  WM: 17.16% | Kits distributed per model proxy for equipment use for activity |
|  | **Vehicles** | None | - | - | - | - | - |
| Recurrent | **Personnel & Per diems – Local** | Local program, administration, and warehouse staff salaries, local consultants, worker compensation fees, local perdiems and travel expenses | Recurrent | 0 | % of distributor days (paid by PSI) | CM: 89%  FM: 0%  WM: 11% | PSI-distributor days per model proxy for PSI work time invested in each model. Note, 100% of distributor days devoted to project. |
|  | **Personnel & Per diems –HQ** | Costs associated with DC staff and Eswatini expat staff, international consultants, international travel per diems and transportation | Recurrent | 0 | % of distributor days (all providers) | CM: 69%  FM: 2%  WM: 29% | Total workdays charged on project, per model (PSI+ volunteers + nurses) proxy for PSI-HQ work time invested in each model |
|  | **Personnel & Per diems – Other Providers** | Transport and lunch fees for volunteers | Recurrent | 0 | % of distributor days (volunteers) | CM: 59%  FM: 3%  WM: 38% | Volunteers reported work days per model proxy for volunteer investment to each model. |
|  |  | Salaries of occupation health nurses | Recurrent | 0 | % of distributor days (nurses) | CM: 2%  FM: 8%  WM: 90% | Nurse reported work days per model proxy for company’s investment to each model. |
|  | **Supplies** | Office supplies (ex: stationary) | Recurrent | 0 | % of kits distributed | CM: 82.71%  FM: 0.13%  WM: 17.16% | Kits distributed per model proxy for supply use for activity |
|  | **Test Kits** | HIV Self-test kits, shipping and handling | Recurrent | 0 | % of kits distributed | CM: 82.71%  FM: 0.13%  WM: 17.16% | Kits distributed per model |
|  | **Vehicle operation, maintenance and transport** | Local transportation, vehicle repairs and maintenance costs, insurance, and fuel. | Recurrent | 0 | % of PSI distributor days | CM: 78.5%  FM: 0.9%  WM: 20.6% | Sum of mileage per model not available. Transportation costs covered in volunteer personnel costs. % of PSI + Company workdays per model used as a proxy for vehicle costs. |
|  | **Building operation and maintenance** | Office and warehouse repairs and maintenance costs, insurance, and utilities | Recurrent | 0 | % of kits distributed | CM: 82.71%  FM: 0.13%  WM: 17.16% | Same as Building and Storage |
|  | **Waste management** | None | - | - | - | - | - |
|  | **Other recurrent** | Dues, fees, subscriptions, bank charges, postage and delivery, communication fees, meetings | Recurrent | 0 | % of kits distributed | CM: 82.71%  FM: 0.13%  WM: 17.16% | Kits distributed per model proxy for communication and meeting fees |
| CM: Community Model, FM: Facility Model, WM: Workplace Model | | | | | | | |
