# Supplementary material for: Costs of distributing HIV self-testing kits in Eswatini through community and workplace models
Source: BMC Infect Dis. 2024 Feb 29;22(Suppl 1):976. doi: 10.1186/s12879-023-08694-y (PMC10902928; doi:10.1186/s12879-023-08694-y)
Supplement: Supplementary file 3 — Additional file 3. Distribution, onsite testing, and reported outcomes. [file 12879_2023_8694_MOESM3_ESM.docx]

**Additional file 3 – M&E Outcomes distribution, onsite testing, interview reported outcomes**

| HIVST Distribution and Primary Client Data per Distribution Model, April 2019 to March 2020 | | | | | | | |
| --- | --- | --- | --- | --- | --- | --- | --- |
|  | | **Community**  **N (%)** | | **Workplace**  **N (%)** | | | **Total** |
| Total # of tests distributed | | 15,864 (83%) | | 3291 (17%) | | | 19,155 |
| Geographic  Hhohho  Lubombo  Manzini  Shiselweni | | 3056 (19%)  1799 (11%)  9582 (60%)  1427 (9%) | | 228 (7%)  313 (10%)  2418 (73%)  332 (10%) | | | 3284 (17%)  2112 (11%)  12000 (63%)  1759 (9%) |
| Total # of clients reached | | 11091 (85%) | | 1932 (15%) | | | 13031 |
| Average # of tests per client | | 1.43 | | 1.70 | | | 1.47 |
| Gender  Female  Male  Transgender  Unknown | | 6068 (55%)  4965 (45%)  2 (0.02%)  56 (0.5%) | | 880 (46%)  1047 (54%)  -  5 (0.26%) | | | 6948 (53%)  6012 (46%)  2 (0.02%)  61 (1%) |
| Age Group  <15  15-19  20-24  25-29  30-34  35-39  40-44  45-49  50+  Unknown | | 6 (0.05%)  1222 (11%)  3194 (29%)  2751 (25%)  1737 (16%)  974 (9%)  566 (5%)  259 (2%)  312 (3%)  70 (0.63%) | | -  48 (2%)  323 (17%)  464 (24%)  417 (22%)  274 (14%)  186 (10%)  97 (5%)  116 (6%)  7 (0.36%) | | | 6 (0.05%)  1270 (10%)  3517 (27%)  3215 (25%)  2154 (17%)  1248 (10%)  752 (6%)  356 (3%)  428 (3%)  77 (1%) |
| Client Population  FSW  MSM  IDU  Gen Pop  Unknown | | 31 (0.28%)  8 (0.07%)  11 (0.10%)  11033 (99%)  8 (0.07%) | | 7 (0.36%)  -  4 (0.21%)  1915 (99%)  6 (0.31%) | | | 38 (0.29%)  8 (0.06%)  15 (0.12%)  12948 (99%)  14 (0.11%) |
| HIV Testing History  Never  t < 2 months  2 months < t < 12 months  t > 12 months  Unknown | | 649 (6%)  40 (0.36%)  6514 (59%)  3150 (28%)  738 (7%) | | 62 (3%)  5 (0.26%)  1226 (63%)  471 (24%)  168 (9%) | | | 711 (6%)  45 (0.35%)  7740 (59%)  3621 (28%)  906 (7%) |
| Onsite HIV Self-Testing Results per Distribution Model, April 2019 to March 2020 | | | | | | | |
|  | | **Community**  **N (%)** | | **Workplace**  **N (%)** | | | **Total**  **N (%)** |
| Total # of clients reached | | 11091 | | 1932 | | | 13023 |
| On/offsite Testing  Did not test on site  Tested on site | | 7929 (71%)  3162 (29%) | | 1784 (92%)  148 (8%) | | | 9713 (75%)  3310 (25%) |
| Onsite Testing Gender  Female  Male  Transgender  Unknown | | 1467 (46%)  1694 (54%)  -  1 (0%) | | 53 (36%)  94 (64%)  -  1 (1%) | | | 1520 (46%)  1788 (54%)  -  2 (0%) |
| Onsite Testing Age Group  <15  15-19  20-24  25-29  30-34  35-39  40-44  45-49  50+  Unknown | | 4 (0.001%)  452 (14%)  1027 (32%)  753 (24%)  419 (13%)  220 (7%)  139 (4%)  68 (2%)  78 (2%)  2 (0%) | | -  3 (2%)  29 (20%)  34 (23%)  30 (20%)  19 (13%)  16 (11%)  4 (3%)  12 (8%)  1 (1%) | | | 4 (0.001%)  455 (14%)  1056 (32%)  787 (24%)  449 (14%)  239 (7%)  155 (4%)  72 (2%)  90 (2%)  3 (0.001%) |
| Onsite Testing HIV Testing History  Never  t < 2 months  2 months < t < 12 months  t > 12 months  Unknown | | 414 (13%)  7 (0.002%)  1313 (42%)  1428 (45%)  - | | 15 (10%)  -  75 (51%)  58 (39%)  - | | | 429 (13%)  7 (0.002%)  1388 (42%)  1486 (45%)  - |
| Onsite Testing Results  Reactive  Non-reactive  Results non-disclosed | | 67 (2%)  3055 (97%)  40 (1%) | | 9 (6%)  136 (92%)  3 (2%) | | | 76 (2%)  3191 (96%)  43 (1%) |
| Results of Follow-up Calls with HIVST Primary Clients | | | | | | | |
|  | **Community**  **N (%)** | | **Workplace**  **N (%)** | | **Unknown**  **N (%)** | **Total**  **N (%)** | |
| Total # of primary clients followed-up | 687 | | 234 | | 1073 | 1994 | |
| Gender (n=1994)  Female  Male  Unknown | 383 (56%)  269 (39%)  35 (5%) | | 100 (43%)  85 (36%)  49 (21%) | | 110 (10%)  128 (12%)  835 (78%) | 593 (30%)  482 (24%)  919 (46%) | |
| Age Group (n=1994)  <15  15-19  20-24  25-29  30-34  35-39  40-44  45-49  50+  Unknown | -  52 (8%)  140 (20%)  186 (27%)  122 (18%)  73 (11%)  32 (5%)  23 (3%)  24 (3%)  35 (5%) | | -  6 (3%)  24 (10%)  43 (18%)  46 (20%)  33 (14%)  17 (7%)  6 (3%)  11 (5%)  48 (21%) | | -  23 (2%)  52 (5%)  53 (5%)  48 (4%)  26 (2%)  20 (2%)  9 (1%)  7 (1%)  835 (78%) | -  81 (4%)  216 (11%)  282 (14%)  216 (11%)  132 (7%)  69 (3%)  38 (2%)  42 (2%)  918 (46%) | |
| Reported use of HIVST kit (n=1994)  Yes  No  NA/No Response | 572 (83%)  29 (4%)  86 (13%) | | 196 (84%)  7 (3%)  31 (13%) | | 591 (55%)  37 (3%)  445 (41%) | 1359 (68%)  73 (4%)  562 (28%) | |
| Reported HIVST result (n=1395)  Reactive  Non-reactive  Unknown/No Response | 24 (4%)  541 (95%)  7 (1%) | | 4 (2%)  186 (95%)  6 (3%) | | 37 (6%)  537 (91%)  17 (3%) | 65 (5%)  1264 (93%)  30 (2%) | |
| Confirmed testing (n=65)  Yes  No  Unknown/No Response | 15 (63%)  1 (4%)  8 (33%) | | 1 (25%)  -  3 (75%) | | 15 (41%)  1 (3%)  21 (57%) | 31 (48%)  2 (3%)  32 (49%) | |
| Confirmed HIV result (n=31)  Positive  Negative  Unknown/No Response | 14 (93%)  -  1 (7%) | | 1 (100%)  -  - | | 13 (87%)  -  2 (13%) | 28 (90%)  -  3 (10%) | |
| Initiated ART (n=28)  Yes  No  NA/No Response | 9 (64%)  2 (14%)  3 (21%) | | 1 (100%)  -  - | | 7 (54%)  2 (15%)  4 (31%) | 17 (61%)  4 (14%)  7 (25%) | |
| Used PrEP (n=1994)  Yes  No  NA/No Response | 2 (0.3%)  475 (69%)  210 (31%) | | 3 (1%)  150 (64%)  81 (35%) | | 7 (0.7%)  377 (35%)  689 (64%) | 12 (0.6%)  1002 (50%)  980 (49%) | |
| Male Circumcision (n=1994)  Yes  No  NA/No Response | 17 (2%)  121 (18%)  549 (80%) | | 4 (2%)  50 (21%)  180 (77%) | | 20 (1%)  167 (16%)  886 (83%) | 41 (2%)  338 (17%)  1615 (81%) | |
| Total # of Secondary clients | 344 | | 135 | | 324 | 803 | |
| Gender (n=803)  Female  Male  Unknown | 196 (57%)  148 (43%)  - | | 78 (58%)  57 (42%)  - | | 150 (46%)  174 (54%)  - | 424 (53%)  379 (47%)  - | |
| Age Group (n=803)  <15  15-19  20-24  25-29  30-34  35-39  40-44  45-49  50+  Unknown | 5 (1%)  35 (10%)  86 (25%)  49 (14%)  84 (24%)  28 (8%)  21 (6%)  7 (2%)  10 (3%)  19 (6%) | | 2 (1%)  6 (4%)  36 (27%)  17 (13%)  32 (24%)  10 (7%)  9 (7%)  3 (2%)  11 (8%)  9 (7%) | | 10 (3%)  18 (6%)  66 (20%)  53 (16%)  71 (22%)  43 (13%)  15 (5%)  5 (2%)  16 (5%)  27 (8%) | 17 (2%)  59 (7%)  188 (23%)  119 (15%)  187 (23%)  81 (10%)  45 (6%)  15 (2%)  37 (5%)  55 (7%) | |
| Relationship to Primary (n=803)  Partner  Biological Child  Family  Associate  Other  Unknown | 158 (46%)  18 (5%)  44 (13%)  112 (33%)  2 (1%)  10 (3%) | | 60 (44%)  13 (10%)  22 (16%)  34 (25%)  -  6 (4%) | | 175 (54%)  32 (10%)  62 (19%)  41 (13%)  2 (1%)  12 (4%) | 393 (49%)  63 (8%)  128 (16%)  187 (23%)  4 (0.5%)  28 (3%) | |
| Reported use of HIVST kit (n=803)  Yes  No  NA/No Response | 205 (60%)  40 (12%)  99 (29%) | | 80 (59%)  25 (19%)  30 (22%) | | 228 (70%)  60 (19%)  36 (11%) | 513 (64%)  125 (16%)  165 (21%) | |
| Reported result of HIVST (n=513)  Reactive  Non-reactive  NA/No Response | 2 (1%)  185 (90%)  18 (9%) | | 1 (1%)  63 (79%)  16 (20%) | | 7 (3%)  207 (91%)  14 (6%) | 10 (2%)  455 (89%)  48 (9%) | |
| Confirmed HIV result (n=10)  Positive  Negative  NA/No Response | 1 (50%)  -  1 (50%) | | -  -  1 (100%) | | 2 (29%)  -  5 (71%) | 3 (30%)  -  7 (70%) | |
| Initiated ART (n=3)  Yes  No  NA/No Response | -  -  1 (100%) | | -  -  - | | -  -  2 (100%) | -  -  3 (100%) | |
| Used PrEP (n=803)  Yes  No  NA/No Response | 2 (0.6%)  160 (47%)  182 (53%) | | -  65 (49%)  70 (52%) | | 2 (0.6%)  164 (52%)  158 (49%) | 4 (0.5%)  389 (49%)  410 (51%) | |
| Male Circumcision (N=803)  Yes  No  NA/No Response | 5 (1.5%)  67 (20%)  272 (79%) | | -  28 (21%)  107 (79%) | | 5 (1%)  112 (35%)  207 (64%) | 10 (1%)  207 (26%)   1. (73%) | |
