# Supplementary material for: Costs of distributing HIV self-testing kits in Eswatini through community and workplace models
Source: BMC Infect Dis. 2024 Feb 29;22(Suppl 1):976. doi: 10.1186/s12879-023-08694-y (PMC10902928; doi:10.1186/s12879-023-08694-y)
Supplement: Supplementary file 4 — Additional file 4. Best-case and worst-case estimates, measurements, and assumptions. [file 12879_2023_8694_MOESM4_ESM.docx]

**Additional file 4 – Best-Case and Worst-Case Estimates, Measurements, and Assumptions.**

|  | **Best-Case** | **Measurement & Assumptions** | **Worst-Case** | **Measurement & Assumptions** |
| --- | --- | --- | --- | --- |
| **Total HIVST kits distributed** | **19,155** | M&E data. Kits indicated as used for demonstration or wasted, removed. | **19,155** | M&E data. Kits indicated as used for demonstration or wasted, removed. |
| **Total primary clients reached** | 13,023 | M&E data | 13,023 | M&E data |
| Testing Rate Estimates | | | | |
| Total onsite tests | 3,310 | M&E data | 3,310 | M&E data |
| Offsite primary clients | 9,713 | Remaining clients not tested onsite | 9,713 | Remaining clients not tested onsite |
| Offsite primary clients tested | 9,218 | 95% testing uptake. Non-responses ignored. | 6,621 | 68% testing uptake. Non-responses included. |
| Offsite secondary clients | 6,132 | Remaining tests not used by primary clients | 6,132 | Remaining tests not used by primary clients |
| Offsite secondary clients tested | 4,931 | 80% testing uptake. Non-responses ignored. | 3,917 | 64% testing uptake. Non-responses included. |
| **Total clients tested** | **17,458** | Sum of onsite and offsite primary and secondary clients tested | **13,848** | Sum of onsite and offsite primary and secondary clients tested |
| Reactivity Rate Estimates | | | | |
| Total onsite reactives | 76 | M&E data | 76 | M&E data |
| Offsite primary reactives | 451 | 4.89% reactivity rate. Non-responses ignored. | 317 | 4.78% reactivity rate. Non-responses included. |
| Offsite secondary reactives | 106 | 2.15% reactivity rate. | 77 | 1.95% reactivity rate. |
| **Total reactives** | **633** | Sum of onsite and offsite primary and secondary clients tested reactive. | **469** | Sum of onsite and offsite primary and secondary clients tested reactive. |
| Confirmed Positives | | | | |
| Onsite clients presenting for confirmatory testing | 76 | M&E data. All onsite reactives assumed to have linked to confirmatory testing. | 76 | M&E data. All onsite reactives assumed to have linked to confirmatory testing. |
| Offsite primary clients presenting for confirmatory testing | 424 | 94% linkage to confirmatory testing. Non-responses ignored. | 151 | 48% linkage to confirmatory testing. Non-responses included. |
| Offsite secondary clients presenting for confirmatory testing | 106 | 100% linkage to confirmatory testing. Non-responses ignored. | 23 | 30% linkage to confirmatory testing. Non-responses included. |
| **Total clients presenting for confirmatory testing** | **606** | Sum of onsite and offsite primary and secondary clients linked to confirmatory testing. | **250** | Sum of onsite and offsite primary and secondary clients linked to confirmatory testing. |
| ART Initiation | | | | |
| Onsite clients initiating ART | 76 | M&E data. All onsite reactives assumed to have linked to ART initiation. | 76 | M&E data. All onsite reactives assumed to have linked to ART initiation. |
| Offsite primary clients initiating ART | 343 | 81% initiating ART. Non-responses ignored. | 83 | 55% initiating ART. Non-responses included. |
| Offsite secondary clients initiating ART | 86 | 81% initiating ART. Non-responses ignored. | 7 | 30% initiating ART. Non-responses included. |
| **Total clients initiating ART** | **505** | Sum of onsite and offsite primary and secondary clients linked to ART initiation. | **166** | Sum of onsite and offsite primary and secondary clients linked to ART initiation. |
