# Supplementary material for: Costs of distributing HIV self-testing kits in Eswatini through community and workplace models
Source: BMC Infect Dis. 2024 Feb 29;22(Suppl 1):976. doi: 10.1186/s12879-023-08694-y (PMC10902928; doi:10.1186/s12879-023-08694-y)
Supplement: Supplementary file 5 — Additional file 5. Average cost per HIVST kit distributed. [file 12879_2023_8694_MOESM5_ESM.docx]

Additional File 5—The average cost per HIVST kit distributed (adjusted to 2020 US dollars) by number of kits distributed and by number of new HIV infections per year, by country

*Lesotho - d'Elbée, 2020; Eswatini - McGee, 2020; Zimbabwe - Mangenah, 2019; Zambia - Mangenah, 2019; South Africa – Mostert, 2020; Zambia - Ahmed, 2018; Malawi - Mangenah, 2019*
