# Supplementary material for: Costs of distributing HIV self-testing kits in Eswatini through community and workplace models
Source: BMC Infect Dis. 2024 Feb 29;22(Suppl 1):976. doi: 10.1186/s12879-023-08694-y (PMC10902928; doi:10.1186/s12879-023-08694-y)
Supplement: Supplementary file 6 — Additional file 6. Average cost per person tested in Eswatini by testing type. [file 12879_2023_8694_MOESM6_ESM.docx]

Additional File 6—Average cost per person tested in Eswatini (adjusted to 2020 US dollars) by number of tests conducted by testing type

*Home: Home-based Testing - Parker, 2015; Mobile: Mobile Testing and Counseling – Parker, 2015; PITC:* *Provider Initiated Testing and Counseling - Obure, 2012; VCT: Voluntary Counseling and Testing - Obure, 2012; HIVST: HIV Self-Testing - McGee, 2020*
